# Assessing Medical Emergency E-referral Request Acceptance Patterns and Trends: A Comprehensive Analysis of Secondary Data From the Kingdom of Saudi Arabia

**DOI:** 10.7759/cureus.53511

**Published:** 2024-02-03

**Authors:** Nawfal Aljerian, Abdullah Alharbi

**Affiliations:** 1 Emergency Medicine, King Saud bin Abdulaziz University for Health Sciences, Riyadh, SAU; 2 Emergency Medicine, Medical Referrals Centre, Ministry of Health, Riyadh, SAU; 3 Family and Community Medicine, Faculty of Medicine, Jazan University, Jazan, SAU

**Keywords:** health care system ksa, smarc, acceptance, emergency, e-referrals

## Abstract

Introduction

Patient transfers in emergencies have been linked to reduced mortality rates and enhanced quality of care. The Saudi Medical Appointments and Referrals Centre (SMARC), an e-referral system in the Kingdom of Saudi Arabia (KSA) since 2019, plays a crucial role in ensuring quality and continuity of care. The findings of this study can provide valuable insights into the effectiveness of the e-referral system and identify potential areas for improvement in the management of emergency cases.

Objective

This study aims to examine e-referral patterns for emergency medical cases throughout all 13 administrative regions of KSA. Concurrently, it estimates the acceptance rate of medical emergency referrals and investigates associated factors among KSA hospitals.

Methods

This retrospective study utilized secondary data from the SMARC e-referral system, specifically focusing on medical emergency e-referral requests in the entire KSA during 2021. Descriptive univariate analyses were conducted to characterize the referral requests, followed by bivariate analyses to explore associations between factors and referral acceptance. Adjusted multiple logistic regression analyses were then performed to calculate adjusted odds ratios (ORs) and corresponding 95% confidence intervals, controlling for potential confounding variables.

Results

A total of 29,660 medical emergency referral requests were initiated across all regions of KSA during the study time frame, and, of these, 20,523 (69.19%) were accepted. The average age of patients with a medical emergency referral was 52 years old, and referral requests were higher among Saudis (13,781; 54.18%), males (13,781; 54.18%), and those from the Western region (10,560; 35.60%). Nearly 20,854 (70%) were due to the unavailability of specialized doctors or specialties in facilities. Based on multi-logistic regression, referral request acceptance was high in some factors as follows: compared to the Central region, requests from the Northern, Southern, Eastern, and Western regions had higher acceptance rates at 123%, 64%, 54%, and 46%, respectively. In addition, referral requests that were due to the unavailability of a specialized doctor or medical equipment had higher acceptance rates (19% and 16%), respectively, than those due to the unavailability of a specific specialty.

Conclusion

This study provides valuable insights into regional variations, sociodemographic factors, and referral reasons within the medical emergency e-referral system in the KSA. By estimating the acceptance rate of medical emergency referrals and investigating associated factors, this analysis confirms the effectiveness of the e-referral system in facilitating access to quality care, particularly for marginalized patients. The study highlights the need for health policy improvements to ensure equitable resource allocation and reduce disparities in healthcare access.

## Introduction

An effective referral system that encourages effective collaboration through all levels of care is a necessity to provide the highest quality healthcare [1]. An effective referral system bridges the gaps in healthcare infrastructure and provides an integrated channel through which healthcare facilities and regional hospitals can work together to ensure that patients have access to all necessary healthcare resources, including those that may not be available at their original treatment location [2,3]. A variety of factors contribute to the success of a referral system, including barriers to patient access, the overall functioning of the healthcare system, communication systems, human and non-human resources, technology, and patient behavior in response to the referral [4].

The electronic referral (e-referral) system in healthcare aims to improve wait times and efficiency by standardizing information and communication electronically. This system enhances the efficiency of the health system through streamlined processes, improved communication, optimized resource allocation, and data-driven decision-making [5]. There are significant consequences for extended durations of stay in the emergency department (ED) and for delayed medical intervention in emergency cases, including increased morbidity and mortality, increased cost, delays in necessary treatment, suboptimal patient outcomes, and diminished quality of care for other patients [6-9]. This study focused on understanding the types of referrals, acceptance rates, and the role of a referral center in coordinating and expediting the referral process to assess the quality and efficiency of a referral system, particularly in emergencies. By examining referral characteristics and acceptance rates of emergency referrals processed through the referral system operated by the Ministry of Health in the Kingdom of Saudi Arabia, this research introduces an innovative approach that provides valuable insights for healthcare systems globally and has the potential to improve healthcare practices worldwide.

Current available literature indicates a significant variation in the rate of referral requests made by physicians, with a notable increase in referral requests in recent years [2,10-13]. This increase in referral requests can be attributed to several factors, including a growing and aging population, and the increased complexity of required care, which requires the involvement of specialized physicians [2,14,15]. While effectively managing the surge of referral requests poses a significant challenge, doing so is crucial to guarantee that patients receive needed care in a timely manner without unnecessary delays in disease diagnosis and treatment.

The KSA is in the midst of a major transformation of its healthcare system, creating a world-class health system for the twenty-first century, as envisioned by the Saudi Vision 2030 [16]. Healthcare reform goals of Vision 2030 include ensuring that all citizens have access to high-quality healthcare, relying on primary care as the foundation of the system, and engaging competition and accountability to create a more efficient system. Under Vision 2030, the existing 13 administrative regions will be consolidated into five business units: Central, Northern, Southern, Eastern, and Western. Each business unit will have its own private holding company that will be responsible for the administration of healthcare in that region [17-19].

In Saudi Arabia, all referral requests throughout the country’s 13 administrative regions are processed through the Saudi Medical Appointments and Referrals Centre (SMARC), the national electronic referral system [2]. Disparities in the accessibility of healthcare services, particularly between different regions of a single country, have been well-documented, and can often be attributed to differences in the availability of hospital beds, qualified physicians, and medical equipment [20,21]. The purpose of an effective referral system is to transfer patients from locations with insufficient resources to facilities that can meet a particular patient’s specific medical needs, thus, SMARC facilitates referrals both within a region and from one region to another [2,22]. This study is the first to examine patterns of e-referrals for emergency medical cases throughout all 13 administrative regions of Saudi Arabia. The study used secondary data obtained through the SMARC referral system from a broad sample of e-referral requests and aimed to identify the predictors affecting the acceptance of e-referral requests. The results of this analysis reveal critical flaws in the e-referral system, indicating a need for changes to ensure that all patients can access needed care within an appropriate time frame.

## Materials and methods

Study design, setting, and data collection and availability

A retrospective statistical analysis was conducted using secondary data collected through the SMARC e-referral system of KSA in all 13 administrative regions of the country. The SMARC, a center in the Ministry of Health, has authorized access to its extensive database that connects healthcare facilities in Saudi Arabia. SMARC includes public and private hospitals, ranging from secondary to tertiary, as well as specialized institutions and others. SMARC is the national authority in Saudi Arabia that is responsible for coordinating and approving medical referrals. To facilitate this process, each hospital in the country has an Office of Coordination and Eligibility for Treatment (OCET), which collaborates with the Unified System of Medical Referrals (USMR). When a physician determines the need for a patient's referral, the request is sent to the OCET, which uploads it to the USMR. Referrals are categorized as lifesaving, emergency, or routine based on the patient's condition. SMARC's team monitors and prioritizes severe cases for prompt referral. This study utilizes retrospective data from SMARC's database, transferred to a Microsoft Excel spreadsheet (Microsoft Corporation, Redmond, WA, USA) for analysis. The dataset comprises coded and non-coded variables, offering insights into referral patterns and healthcare utilization. We focus on medical emergencies requiring transfers within 24 hours, as SMARC defines [2].

Measurements

The study focused specifically on emergency medical cases in 2021, and data obtained included the administrative referral area, reason for referral, whether the referral was accepted or rejected, as well as sociodemographic variables such as age, nationality, and sex. While the KSA healthcare system was divided into 13 administrative areas at the time of the data collection in 2021, the system is now consolidated into 5 new Ministry of Health business units, which are as follows: the Southern BU (SBU) includes Asir, Jazan, and Najran; the Northern BU (NBU) includes Al Jawf, Hail, Northern Frontier, and Tabuk; the Central BU (CBU) includes Riyadh and Al Qassim; the Western BU (WBU) includes Makkah, Medina, and Al Baha; and the Eastern BU (EBU) includes Sharqiyah [17].

Ethical considerations

Ethical approval was received from the Ministry of Health institutional review board (IRB log No:23-77 E). All data used in the study were completely anonymized to remove all patient-identifying information. Stringent measures were implemented to ensure the data were handled with appropriate security and protection. The data were used solely for the specific research objectives and all research was conducted in accordance with ethical guidelines and regulations.

Statistical analysis

The main objective of the study was to determine which factors were correlated with the acceptance or rejection of emergency medical referrals. Data analysis occurred in three steps. First, descriptive statistics, including proportions and frequencies, were used to analyze the data. Second, cross-binary tabulation tests were performed with chi-square tests to study the relationships between acceptance status and possible predictors. Finally, adjusted multiple logistic regression analysis was performed to calculate both the odds ratios (ORs) and the corresponding 95% confidence levels (CIs), with a significance level set at 0.05. All statistical analyses were conducted using Stata Statistical Software Version 16 (College Station, TX: StataCorp LLC).

## Results

Table 1 presents the characteristics of emergency medical e-referrals in Saudi Arabia in 2021. The table reports the total number of e-referral requests (29,660), the mean age of patients referred (52.99 years, SD=20.97), and the distribution of referrals by gender (males (13,781; 54.18%) and females (11,653; 45.82%)) and nationality (Saudi (25,141; 84.76%) and non-Saudi (4,519; 15.24%)). It also provides information on the regions of business units involved in the referrals (Central, Eastern, Western, Northern, and Southern). The most common reasons for referral were the unavailability of subspecialty (15,776; 53.19%), physician (5,078; 17.12%), machine (3,343; 11.27%), and bed (3,4109; 11.50%). Referrals related to social issues occur when patients encounter emergencies at a distance from family, prompting a preference for local hospital referrals to facilitate support and maintain family proximity during critical times, which accounted for a small proportion, 54 (0.18%), while health crises represented 1,999 (6.74%) of the total.

**Table 1:** Emergency medical e-referral status and characteristics in the Kingdom of Saudi Arabia (2021) SD: standard deviation; BU: business unit

Referral characteristics	Total N (%): 29,660 (100%)
Age (Mean, SD), Years	52.99 (20.97%)
Sex	
Males	13,781 (54.18%)
Females	11,653 (45.82%)
Nationality	
Non-Saudi	4,519 (15.24%)
Saudi	25,141 (84.76%)
Regions of Business Units	
Central BU	3,038 (10.24%)
Eastern BU	3,764 (12.69%)
Western BU	10,560 (35.60%)
Northern BU	4,754 (16.03%)
Southern BU	7,544 (25.43%)
Reasons for Referral	
Unavailable subspecialty	15,776 (53.19%)
Unavailable physician	5,078 (17.12%)
Unavailable machine	3,343 (11.27%)
Unavailable bed	3,4109 (11.50%)
Social	54 (0.18%)
Health crisis	1,999 (6.74%)

Table 2 examines the distribution of medical emergency e-referrals based on the status of the referral requests in 2021. The table compares rejected cases to accepted cases and provides corresponding p-values. The average age of patients in rejected and accepted cases was similar, with no statistically significant difference. The distribution of male and female patients was also similar between rejected and accepted cases. However, there was a significant difference in the distribution of referrals across different regions. Northern and southern regions had a higher proportion of accepted cases among regions: 3658 (76.95%) and 5345 (70.85%) of cases were accepted, respectively. For the reason of referral and acceptance status, the most commonly accepted reason was the unavailability of doctors, followed by the unavailability of the required machine in 3697 (72.80%) and 2397 (71.70%), respectively.

**Table 2:** Bivariate analysis of accepted status and medical emergency e-referral associated factors in the Kingdom of Saudi Arabia (year 2021) BU: business unit; KSA: Kingdom of Saudi Arabia; *chi-square test; #p-value based on the one-way analysis of variance (ANOVA) test. * Statistical significance at P < 0.05 (significant p-values are in bold)

	Rejected: 9,137 (30.81%)	Accepted: 20,523 (69.19%)	P-value*
Referral characteristics			
Age years (Mean, SD), Years	53.23 (20.92%)	52.88 (21.00%)	0.67^#^
Sex			0.72
Males	4282 (31.07%)	9499 (68.93%)	
Females	3645 (31.28%)	8008 (68.72%)	
Nationality			0.23
Non-Saudi	1426 (31.56%)	3093 (68.44%)	
Saudi	7711 (30.67%)	17430 (69.33%)	
Regions of Business Units			0.00*
Central BU	1249 (41.11%)	1789 (58.89%)	
Eastern BU	1196 (31.77%)	2568 (68.23%)	
Western BU	3397 (32.17%)	7163 (67.83%)	
Northern BU	1096 (23.05%)	3658 (76.95%)	
Southern BU	2199 (29.15%)	5345 (70.85%)	
Reason for Referral			0.00*
Unavailable subspecialty	5070 (32.14%)	10706 (67.86%)	
Unavailable physician	1381 (27.20%)	3697 (72.80%)	
Unavailable machine	946 (28.30%)	2397 (71.70%)	
Unavailable Bed	1087 (31.88%)	2323 (68.12%)	
Social	18 (33.33%)	36 (66.67%)	
Health crisis	635 (31.77%)	1364 (68.23%)	

Table 3 presents the results of the adjusted multivariable associations between various predictors and referral acceptance. The analysis found that age, sex, and nationality were not significantly associated with the acceptance rate of referrals. Regarding the business units (BUs), the regions under the northern BU demonstrated more than double the likelihood of referral acceptance compared to central BU (adjusted odds ratio (OR) = 2.23, 95% CI= 2.01 - 2.48). The other BUs also showed a higher likelihood of acceptance compared to the Central BUs, albeit to a lesser extent. When the reasons for referral were compared to the acceptance rate, the analysis showed that ER requests citing a lack of specialist doctors or a lack of a machine had a statistically significant higher likelihood of being accepted than the department's unavailability.

**Table 3:** Multivariable associations of predictors for referral acceptance aOR: adjusted odds ratio; CI: confidence interval * Statistical significance at P < 0.05 (significant p-values are in bold)

Acceptance Status	AOR	p-value	95% CI
Age		0.46	0.99 - 1.01
Sex			
Male	1		
Female	0.98	0.64	0.93 -1.04
Nationality			
Non-Saudi	1		
Saudi	1.01	0.83	0.93 -1.08
Regions			
Central Regions	1		
Eastern Regions	1.54	0.00*	1.38 -1.71
Western Regions	1.46	0.00*	1.34 -1.60
Northern Regions	2.23	0.00*	2.01 - 2.48
Southern Regions	1.64	0.00*	1.49 -1.80
Reasons for Referral			
Unavailable Subspecialty	1		
Unavailable Specialized Doctor	1.19	0.00*	1.10 - 1.29
Unavailable Machine	1.16	0.00*	1.06 - 1.28
Unavailable Bed	1.02	0.52	0.94 - 1.12
Social	0.99	0.98	0.54 - 1.81
Health Crisis	1.02	0.61	0.92 -1.14

## Discussion

This study aimed to determine the acceptance rate of e-referral requests and to identify predictive factors for acceptance in medical emergency cases, utilizing secondary data collected across the KSA. This study was the first to estimate the prevalence and associated predictors for medical emergency e-referral requests. Approximately 20,523 (69.19%) of all medical emergency referrals were accepted, with those citing a lack of available specialists or lack of available medical equipment most likely to be accepted. The region with the highest rate of acceptance was the Northern BU. No significant association was found between acceptance and age, sex, or nationality.

Electronic referral (e-referral) systems facilitate part of the referral workflow through IT. They have been tested in Australia [23], utilized by the US Veterans Administration, which faced challenges with e-referral coordination due to policy and standardization issues [24], and assessed in the UK, showing mixed results on referral content quality [25]. In Denmark, e-referrals are widespread, used by all GPs for around 40% of referrals, and yielding significant cost savings [26]. Norwegian experiences emphasize the need for cooperation between referring clinicians to ensure successful e-referral processes [27]. Warren et al.'s study observed a significant and rapid voluntary adoption of e-referrals, leading to quicker, more dependable, and transparent referral processing in New Zealand [28]. Current research on referral patterns largely focuses on primary healthcare [29-31], unlike the SMARC system in the KSA, which deals exclusively with secondary, tertiary, and specialized care. However, the studies mentioned above yield several other vital insights. Still, none of the studies on referral systems have been evaluated for their distribution in emergency settings, which is the focus of our research.

The study identified the mean age for medical emergency e-referrals to be 52 years old, consistent with previous research findings of 25-45 years being the most common age group for emergency visits [32]. Both studies had an age distribution that reflects the general population of Saudi Arabia, with a majority of individuals on the younger end of the age spectrum. This study focused on men seeking emergency room (ER) referrals, in alignment with previous national and international research findings that demonstrate that males are more likely to visit the ER than females [32-34]. Other research has indicated that gender was significant as an effect modifier, primarily in males, indicating a synergistic relationship between gender and chronic disease [34]. Of note, this study focused specifically on referral requests from ER visits rather than overall ER visits.

This finding serves to clarify the increased frequency of men compared to women in our study. Study findings also indicate that there were more referrals for Saudi patients rather than non-Saudi patients, which is in alignment with the overall numbers of Saudis and non-Saudis within the KSA [35]. The study found regional disparities in both the initiation of referral requests and the utilization of referral services. The Western region had the highest percentage of all medical emergency e-referral requests, 10,560 (35.60%) of all requests, which may be attributed to the significant number of visitors this region receives annually. In 2021, approximately 3.8 million foreigners visited Makkah for Hajj and Umrah [36]. The predominant reason for medical emergency e-referral requests in Saudi Arabia was the unavailability of services or specialized doctors, which accounted for nearly 20,854 (70%) of cases. Furthermore, approximately 6,753 (22%) of referrals were attributed to the unavailability of non-human resources, including beds and machines. Regional healthcare services and resource disparities, such as hospitals, physicians, and equipment, are widely acknowledged internationally [20,21].

Regional disparities in healthcare services and resources, including hospitals, equipment, and physicians, have been well-documented worldwide [21]. While studies have documented the challenges inherent in measuring quality through healthcare utilization rates [18], multiple studies have attempted to address the variation in healthcare service quality across different countries, cities, and hospitals [21,37-39]. This study found variations in the acceptance rate of medical emergency e-referral requests across different Ministry of Health BUs, with higher acceptance rates found in the Northern BU, as compared to the central unit, a finding that is consistent with previous studies measuring quality variation during the coronavirus disease 2019 (COVID-19_ pandemic [17-19,40]. These results indicate that the Northern region may have a greater need for improved infrastructure to alleviate resource constraints and address higher demand. Finally, this study demonstrates that shortages of specialized physicians and medical equipment are the most significant predictor of e-referral acceptance [39]. Medical equipment availability is an ongoing concern globally, as complex equipment requires maintenance, training, and eventual replacement [39]. Equipment availability is a significant concern, as lack of access to medical equipment has been associated with adverse outcomes and increased mortality [41].

Strength and limitation

This study has a high degree of validity, given that the SMARC dataset provides nationally representative, extensive, and detailed information, allowing for comprehensive analysis of public health and health services research. This study was the first to evaluate medical emergency e-referral acceptance and rejection rates and potential predictors, and it highlights the importance of having a national system to monitor patient access to healthcare and to identify areas of improvement. The study has two notable limitations. First, the cross-sectional design of the study does not allow for the establishment of causal relationships between referral request acceptance status and associated factors. In addition, the depth of analysis is limited by a lack of access to diagnosis and details of illness severity in the data.

## Conclusions

This study demonstrates that a significant number, approximately 20,523 (69.19%), of medical emergency e-referral requests are accepted in the KSA, with the highest acceptance rates in the Northern and Southern regions and for referrals due to limited access to specialized doctors and equipment. This study highlights the importance of evaluating resource allocations to effectively manage the substantial volume of referrals and the diversity in referral characteristics to minimize the number of referral requests needed and increase acceptance rates for those needed referrals. This process will improve the overall efficiency and effectiveness of the e-referral system.
